# Clinical Heterogeneity in ME/CFS. A Way to Understand Long-COVID19 Fatigue

**DOI:** 10.3389/fpsyt.2021.735784

**Published:** 2021-10-11

**Authors:** Iñigo Murga, Larraitz Aranburu, Pascual A. Gargiulo, Juan Carlos Gómez Esteban, José-Vicente Lafuente

**Affiliations:** ^1^LaNCE-Neuropharm Group, Department of Neuroscience, University of the Basque Country (UPV-EHU), Leioa, Spain; ^2^Department of Mathematics, University of the Basque Country (UPV-EHU), Leioa, Spain; ^3^Lab Experimental Psychology, Consejo Nacional de Investigaciones Científicas y Técnicas (CONICET), Department of Pathology, Universidad Nacional de Cuyo (UNC), Mendoza, Argentina; ^4^Neurodegenerative Disease Group, Biocruces Research Institute, Barakaldo, Spain

**Keywords:** Myalgic Encephalomyelitis, Chronic Fatigue Syndrome, post-viral fatigue, long COVID-19, dysautonomia

## Abstract

The aim of present paper is to identify clinical phenotypes in a cohort of patients affected of Myalgic Encephalomyelitis/Chronic Fatigue Syndrome. Ninety-one patients and 22 healthy controls were studied with the following questionnaires, in addition to medical history: visual analogical scale for fatigue and pain, DePaul questionnaire (post-exertional malaise, immune, neuroendocrine), Pittsburgh sleep quality index, COMPASS-31 (dysautonomia), Montreal cognitive assessment, Toulouse-Piéron test (attention), Hospital Anxiety and Depression test and Karnofsky scale. Co-morbidities and drugs-intake were also recorded. A hierarchical clustering with clinical results was performed. Final study group was made up of 84 patients, mean age 44.41 ± 9.37 years (66 female/18 male) and 22 controls, mean age 45 ± 13.15 years (14 female/8 male). Patients meet diagnostic criteria of Fukuda-1994 and Carruthers-2011. Clustering analysis identify five phenotypes. Two groups without fibromyalgia were differentiated by various levels of anxiety and depression (13 and 20 patients). The other three groups present fibromyalgia plus a patient without it, but with high scores in pain scale, they were segregated by prevalence of dysautonomia (17), neuroendocrine (15), and immunological affectation (19). Regarding gender, women showed higher scores than men in cognition, pain level and depressive syndrome. Mathematical tools are a suitable approach to objectify some elusive features in order to understand the syndrome. Clustering unveils phenotypes combining fibromyalgia with varying degrees of dysautonomia, neuroendocrine or immune features and absence of fibromyalgia with high or low levels of anxiety-depression. There is no a specific phenotype for women or men.

## Introduction

Myalgic Encephalomyelitis (ME) was proposed by Acheson as the name for a group of epidemic outbreaks that took place between 1945 and 1955 in different countries. The symptoms presented by these patients were similar to those shown by people suffering from poliomyelitis and included: fatigue, depression, muscle weakness, headaches and paresthesia, among others (1). Similar clinical presentations have appeared recently in descriptions of patient outcomes some months after suffering from COVID-19 (2). Similar descriptions can be found in the literature under names such as epidemic neuromyasthenia, vegetative neuritis, post-viral fatigue syndrome, raphe nucleus encephalopathy, chronic mononucleosis syndrome or exertion intolerance, among others (3). These conditions can arise in isolated form, but generally appear within the context of epidemics (4).

ME normally begins with a viral infection affecting the respiratory system or gastrointestinal tract, which is followed by severe, persistent “central” fatigue accompanied by headaches, vertigo, muscle weakness, sleep disorders, paresthesia, dysautonomia, blurred vision, diplopia, anosmia, ataxia, emotional problems, etc. (5). Physical examinations, laboratory tests (including cerebrospinal fluid) and brain Structural Magnetic Resonance imaging are in general normal or unspecific (6–8).

At present some of the patients suffering from Long-COVID19 are showing similar clinical profiles to those described above (2, 9, 10). It would therefore be interesting to perform this specific condition, but also to identify some of the after-effects that might also be an in-depth analysis of a group of patients affected by ME, not only to find out more about sequelae of the current SARS CoV-2 pandemic. This virus (RNA-virus) can cause exaggerated immune responses, which can lead to extreme “central” fatigue as occurs in ME. These presentations also include a reduction in B lymphocytes, increasing pro- inflammatory cytokines, neural inflammation and activation of self-reactive T-cells (11).

One of the main problems for ME diagnosis (6) is its clinical heterogeneity and its numerous associated co-morbidities. Nomination and diagnostic criteria have been recently reviewed, although it remains unclear whether they are phenotypes of the same pathology or different diseases (12, 13).

The current study presents an extensive and precise analysis of a series of patients diagnosed with ME, also known to the scientific community as Chronic Fatigue Syndrome (CFS) expressing itself in the literature in a dual terminology (ME/CFS), in order to identify phenotypes (homogeneous subgroups), which would help us gain a better understanding of the syndrome, and define biomarkers. Matching this syndrome to Long-COVID19 pathology we are going to obtain valuable information about the latter condition in different phenotypes of patients affected by COVID19, helping to prepare us for the challenges that lie ahead.

## Materials and Methods

This is a retrospective, observational, analytical case-control study with 91 patients and 22 control subjects. During the course of the study, seven patients withdrew; one after being diagnosed of multiple sclerosis (man) and another after beginning treatment with Rituximab (woman). Of the other five patients who abandoned the study, two are severely worse and three did not indicate why they had withdrawn. The final groups contained 84 cases (66 women/18 men) and 22 control subjects (14 women/8 men).

Inclusion criteria: (1) Provide a report from an authorized physician with a formal diagnosis of Myalgic Encephalomyelitis (ME)/Chronic Fatigue Syndrome (CFS). (2) Aged between 18 and 68. (3) Ethnic group - Caucasian. Exclusion criteria: patients who were unable to take part in the clinical sessions, fill out the proposed questionnaires or were already participating in another study. Other exclusion criteria included: pregnancy, breastfeeding, mitochondrial disease, morbid obesity, major surgery, treatment with chemotherapy, radiotherapy, immunotherapy and systemic steroids during the research period, drug or alcohol abuse in the past or at present, a history of cancer or of cranio- cervical or spinal column surgery.

The control subjects have not suffered from relevant diseases in the past and remain healthy at present. They had to comply with the same exclusion criteria as the patients.

The study was approved by the Ethics Committee for Research in Human Beings (CEISH - University of the Basque Country: act 80/2016 and 114/2019). Participants were recruited through various patient's associations. Volunteers received an information sheet and the informed consent form. Once they had signed the consent form, clinical sessions were performed in such a way as to allow all the information to be gathered by the same research physician.

The patients provided a medical diagnosis of ME and/or CFS without indicating the criteria used. Both concepts are comparable for Spanish clinicians. The research team subsequently re-evaluates them with the international consensus criteria: CFS (Fukuda- 1994) and ME (Carruthers- 2011). In addition, patients supply a basic blood and urine analysis and an electrocardiogram. All these complementary studies remain inside normal parameters. A clinical protocol was applied including the medical history and the following questionnaires: VAS-scale (fatigue, pain), DePaul questionnaire (post-exertional malaise; PEM, immune, neuroendocrine), Pittsburgh (sleep), COMPASS-31 (dysautonomia), Montreal cognitive assessment (MoCA- cognition), Toulouse-Piéron (attention), Hospital Anxiety and Depression test (HAD), and Karnofsky scale. We also gathered information about relevant comorbidities and medicines taken regularly.

### Statistical Analysis

In order to conduct a proper analysis of the quantitative variables, the normality tests Kolmogorov-Smirnov (>50) or Shapiro-Wilks ( ≤ 50) were performed. Once these tests had been conducted, the equality of the averages in the groups were tested for those variables considered as normal. For those not considered as normal, the U the Mann-Whitney test was applied. These were considered to be statistically significant when there was a probability of <5% (*p* < 0.05).

To define different phenotypes in ME/CFS patients were carried out the Gower's distance (14) and the Ward's method or Ward's minimum variance, a criterion applied to hierarchical cluster analysis (15). They are very suitable tools because of the good results reported in the literature and because it does not require patterns or pre-established decisions. Even so, this analysis is closely linked to the distance measure considered when measuring the proximity between patients and to the way the data are collected. Therefore, the first important step in generating the groups was the pre-processing of the database. The following criteria were applied:

1. We considered the ordered qualitative nature of the variables. Thus, it is understood that, they can be represented by numbers but the scales must be homogenized so that when measuring distances, the variables have the same weight. The scales used are 0, 1, 2, and 3 (no record, mild, moderate, and severe, respectively) for all variables of this type.
2. To avoid the duplicity of information, and after verifying that the variables “Anxiety” and “Depression” had a very high correlation, it was decided to unify both variables with the following scale: 0, 1, or 2 (neither of the two pathologies is present, at least one of them has high values or both are very present in the patient).
3. Age has been considered quantitative.
4. The variable “Fibromyalgia” has been treated as a binary variable and, therefore, it has to be introduced as a qualitative variable in the analysis.

Given that there are both qualitative and quantitative variables, it was decided to use Gower's similarity measure (14) as a measure of proximity. Both nearest neighbor and farthest neighbor methods have been tested when deciding the distances between clusters, but the best results (with a clearer separation between clusters) have been obtained using Ward's method, also known as the minimum variance method (15).

All these tests were conducted using the R-Studio Program Version 0.99.489.

## Results

The average age of people suffering ME/CFS was 44.41 ± 9.37 years, while for the control group it was 45 ± 13.15. There were 66 women patients and 18 men, a ratio of almost 4 to 1 (Table 1). The age of diagnosis was around 40 years for both genders. One woman was diagnosed at the early age of 12.

**Table 1:** Data of the tests used in the clinical evaluation.

**Variable**	**ME/CFS *N* = 84 μ(σ) range**	**Control *N* = 22 μ(σ) range**	***p***	**ME/CFS**		
				**Women *N* = 66 μ(σ) range**	**Men *N* = 18 μ(σ) range**	***p***
Age^years^	44.41 (9.37) 18–61	45 (13.5) 18–64		45.48 (8.69) 18–61	40.5 (10.64) 18–58	
Gender	78.57% (Female) 21.42% (Male)	63.63% (Female) 36.36% (Male)		66	18	
Ethnicity	Caucasian	Caucasian		Caucasian	Caucasian	
BMI^(kg/m2)^	24.30 (5.32) 14.68–39.38	23.41 (3.03) 19.53–33.91		24.68 (5.59) 14.68–39.38	22.89 (3.84) 17.44–35.29	
Evolution^years^	5.10 (4.17) 1–17	—		5.39 (4.23) 1–17	4.05 (3.74) 1–13	
Fatigue^VAS^	74.82 (9.61) 50–90	2.72 (18.44) 0–70	^***^	75.53 (8.53) 50–90	72.22 (12.49) 50–90	
Post-exertional malaise^DePaul^	12.01 (3.06) 4–20	0.68 (1.14) 0–5	^***^	12.25 (3.05) 4–20	11.11 (2.92) 7–16	
Pain^VAS^	54.03 (24.57) 0–100	8.40 (14.64) 0–50	^***^	58.16 (21.40) 0–100	38.88 (29.08) 0–85	^*^
Sleep^Pittsburgh^	12.67 (4.67) 1–21	4.54 (3.15) 1–16	^***^	12.63 (4.62) 3–21	12.83 (4.84) 1–19	^*^
Dysautonomia^COMPASS−31^	34.27 (9.89) 12–59	8.77 (4.66) 0–18	^***^	35.46 (9.43) 18–59	29.88 (10.30) 12–49	^**^
Cognition^MoCA^	25.76 (2.45) 18–30	27 (2.33) 21–30	^*^	26.06 (2.26) 18–30	24.66 (2.80) 18–29	^*^
Attention^Toulouse−Pié*ron*^	1.94 (0.80) 0–3	3.04 (0.76) 1 - 5	^***^	1.98 (0.76) 0 - 3	1.77 (0.91) 0 - 3	
Immune^DePaul^	6.97 (3.75) 0 - 18	0.68 (1.36) 0–6	^***^	7.15 (3.65) 0–17	6.33 (4.02) 0–18	^**^
Neuroendocrine^DePaul^	12.58 (5.65) 0–25	1.63 (2.53) 0–9	^***^	12.89 (5.55) 0–25	11.44 (5.88) 0–25	^**^
Anxiety^HADS^	8.5 (4.25) 1–20	4.31 (2.05) 1–9	^***^	8.56 (4.29) 1–20	8.27 (4.06) 1–14	
Depression^HADS^	9.30 (3.94) 2–20	1 (1.67) 0–7	^***^	9.22 (3.85) 3–20	9.61 (4.24) 2–16	
Functionality^Karnofsky^	65.83 (9.15) 50–90	100 (0)	^***^	65.90 (9.37) 50–90	65.55 (8.31) 50–80	
*n* (%) Comorbidity	50 (59.52%) Fibromyalgia	—		42 (63.63%) Fibromyalgia	8 (44.44%) Fibromyalgia	
Daily drugs	2.38 (1.68) 0–6	—		2.4 (1.58) 0–6	2.27 (2.02) 0–6	

* *p < 0.05*.

** *P < 0.01*.

*** *P < 0.001*.

*U de Mann-Whitney*.

*t-Student: Anxiety- ME/CFS vs. control. Age, Dysautonomia, Neuroendocrine, Anxiety and Depression- ME/CFS female vs. male*.

The standard profile of the ME/CFS patients was a woman of between 35 and 51 years old (65.15%) of normal weight (48.48%) - only 15.15% presented with obesity - (Table 1), with higher education (43.93%), a partner (69.69%) and children (59.09%), and with no toxic habits of any kind. As for men, the most frequent age group was also from 35 to 51 years old (61.11%), 72.22% were normal weight and only 5.55% were obese. 33.33% had higher education, 50% had a partner and 72.22% had children. They had no toxic habits.

The patients in this group have been suffering from the syndrome for between 1 and 17 years, with an average of 5.39 ± 4.23 years for women and 4.05 ± 3.74 for men. The average time from the onset of a significant reduction in activity until the moment of diagnosis was 5.24 ± 6.49 years for women and 3.38 ± 3.94 for men. Differences were not statistically significant in any of the cases (Table 1).

All clinical variables show high significant differences respecting to the control group (Table 1) excepting for cognitive functions.

Fatigue took hold progressively over a period of months or years in 75% of the patients, most of them (52.38%) did not know the “cause” of their condition; although 28.57% indicated that chronic stress had played a crucial role. Infectious factors were cited by 15.47% of the group. The clinical evolution of the pathology over time showed a clear tendency toward a progressive worsening in both genders (women - 74.24% and men - 44.44%). There were no significant differences between women and men in any of these parameters (Table 1).

Women had a significantly higher perception of pain than men (*p* = 0.013) when this variable was quantitatively analyzed; however, when it was grouped into three categories: slight, moderate and severe, both genders perceived the pain as moderate (38%) or severe (39%). Only a small number of patients (four men and one woman) felt no pain. Thus, the most frequent co-morbidity was Fibromyalgia (women - 63.63% and men - 44.44%) and other kinds of pain including trochanteric bursitis, sacroiliitis, spondylitis, chronic tendinitis, arthrosis or disc hernia. The most used drugs were non-steroidal anti-inflammatories, anxiolytics and anti-depressants. Most patients present 3-4 co-morbidities and only 7% have none at all (Table 1).

Most of the patients reported poor quality of sleep (92.85%), only six (two men and four women) said they slept well. Three people suffered from apnea as a comorbidity. None of the others offered any medical explanation for their poor-quality sleep (Table 1).

Almost all the participants in this study reported dysautonomic symptoms such as orthostatic intolerance (dizziness), vasomotor (changes in skin color), secretomotor (glandular dryness) or gastrointestinal disorders (diarrhea or constipation), as well as bladder control or pupillary light reflex control (discomfort when exposed to light). All these symptoms were picked up initially by the COMPASS-31 questionnaire. In general, women obtained higher average scores than men in glandular, gastrointestinal and pupillary symptomatology, while the results for vasomotor disorders or bladder control were similar for both genders. Orthostatic intolerance syndrome appeared more frequently in men (33.33%) than in women (15.15%) (Table 1).

Cognitive functions remained within normal levels, with only five patients (two men and three women) showing slight cognitive deterioration. Women obtained significantly better scores than men in the MoCA test (*p* ≤ 0.05). However, when sustained attention was assessed using the Toulouse-Piéron test, generalized low scores were obtained in the Global Attention and Perception Index (GAPI), with no differences between genders (Table 1).

Some slight neuroendocrine manifestations such as: sweaty hands, night sweats, cold extremities, shivering or shaking, feeling cold and/or hot for no reason, a sensation of high or low body temperature, were experienced by 65.47% (*N* = 55). A small number showed no symptoms of this kind (three women and one man). Scores were higher in women. The most frequent endocrine comorbidity was primary hypothyroidism (16.66%, *N* = 14; 13 women and one man) (Table 1).

The HAD questionnaire revealed that approximately one third of the group suffered from anxiety (32.14%, *N* = 27; 20 women and seven men) and depression (36.9%, *N* = 31; 23 women and eight men). No statistically significant differences could be found between genders in the overall scores. Only a small percentage of the patients accepted onto the study provided a medical diagnosis of non-psychotic reactive major depression (14.28%, *N* = 12; 11 women and one man) (Table 1).

The functional autonomy of patients was assessed using the Karnofsky scale. Most patients (88.10%) could not work and needed occasional help in their daily lives (70–50 points). Levels of functionality were identical for both genders. Only 10 participants (nine women and one man) had scores of 90–80 points, and were able to work with some exertion, in spite of suffering symptoms on a daily basis (Table 1).

**Figure 1:**
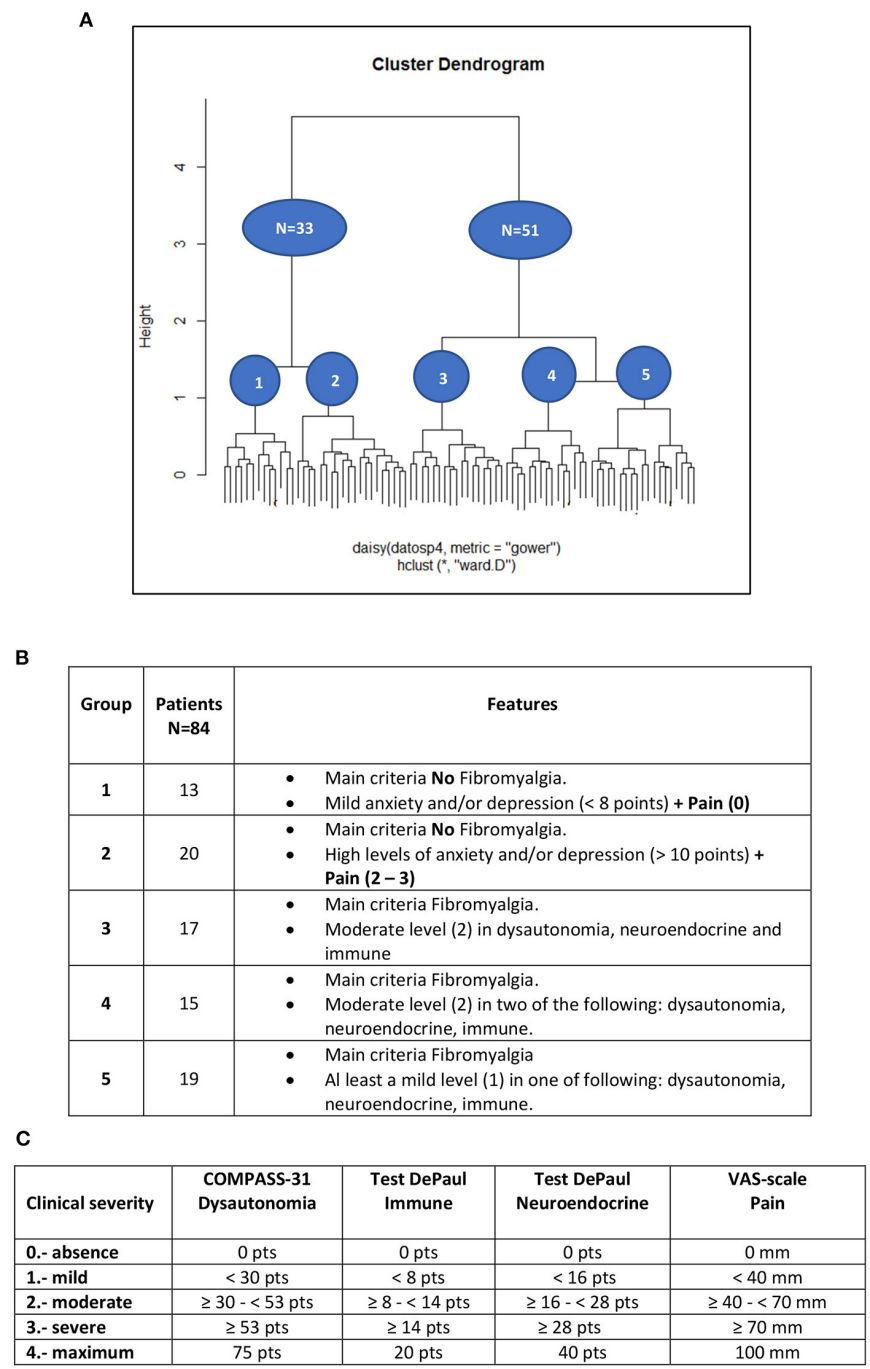
**(A)** Dendrogram displaying cluster analysis of ME/CFS patients. **(B)** Table explaining the features of groups. The scores of Anxiety and Depression test (HAD) establish no repercussion (<8 points) up to 8 borderline (8–10 points) and repercussion (>10 points). **(C)** Table summarizing ranks of the scores for the more relevant tests in the clustering analysis.

Five phenotypes were identified by the clustering analysis. Two corresponded to people without Fibromyalgia (*N* = 33), which can be differentiated by the anxiety and depression levels measured by the HAD test, in which 13 patients showed low scores (<8) and 20 had high scores (>10). The other three phenotypes belonged to the main group of patients with Fibromyalgia (*N* = 50) including one without Fibromyalgia but with a high score in the visual analogic scale for pain and borderline values for the HAD scale (between 8 and 10 points). These 51 patients showed, in general, mild values for HAD and can be grouped according to their symptomatology for dysautonomia, neuroendocrine and immune features. Subgroup 3 (17 patients) showed moderate features in all three domains, sub-group 4 (15 patients) in just two of them and subgroup 5 (19) in just one and sometimes mild or quite low (Figure 1).

## Discussion

The incidence in the present group was of four women to each man, in line with the data reported by other authors (16, 17). It is interesting to note that Long-COVID19 is also more frequent in women than in men, and is also most common in the same age group (around the forties) (18).

Prevalence of ME/CFS seems to increase from puberty onwards in women, but not in men. In adolescence, this illness is already 2–3 times more common in women than in men (19). There are various possible reasons for this, but it is perhaps worth highlighting that differences in reactivity between genders regarding stress have been extensively documented (20, 21), for instance in studies about the role of the Bed Nucleus of the Stria Terminalis (BNST) as a modulator in dysregulation of mood, anxiety and fear. This sexually dimorphic nucleus exerts these functions firstly, through its connection with essential emotional processing regions, such as the prefrontal cortex, the hippocampus and the amygdala and secondly, *via* the paraventricular nucleus triggering the stimulation of the hypothalamic-pituitary-adrenal axis (22).

Delays in patient diagnosis have an unfortunate collateral effect, in that they tend to blur the patient's memory of how the illness started. This information is of enormous interest for categorizing clinical subgroups according to how the illness started (suddenly vs. latently), post-infection, post-stress, etc. (13). Hence, the relevance of analyzing similarities to Long-COVID19, as for the first time it is possible to analyze the early stages of a post-viral central fatigue with modern techniques, for instance using MRI-PET to detect and evaluate neuro-inflammation (23). In our series, most patients did not know what might have triggered their illness, 28.57% suggested chronic stress and 15.47% pointed to an earlier infectious episode. Authors like Chu et al. (24) reported that the most common etiologies were infections (64%; of which 90% were viral, mainly Epstein Barr) followed by extremely stressful events (39%). This may also be related with the neuro- inflammatory hypothesis put forward by Jason et al. (25), according to which a chronic sub- threshold stimulus or a high-intensity acute stimulus could induce hypersensitivity and trigger the typical signs and symptoms of this syndrome.

Fatigue, defined as a reduction of at least 50% of normal activity, is a severe condition that is perceived in the same way by both genders. This condition came on progressively (75%) over the course of months or years in our group, a finding that contrasted with Arruti et al. (26), who found that the illness began acutely after convalescence from an infectious agent (e.g., mononucleosis or herpes).

When asked about the evolution of their illness, patients described it as a process of continuous worsening (67.85%). This contrasts with the fluctuating nature of the illness described by Stoothoff et al. (27) in their series. Post-exertional malaise (PEM) implies a worsening of the symptoms in response to minimal physical or mental effort. Patients affected of ME/CFS frequently report the persistence of this malaise for more than 24 h, with periods of partial or total recovery that continue over several days. This feature distinguishes ME/CFS from other neurological pathologies, which also present “central” fatigue, such as Multiple Sclerosis or post-Polio Syndrome (28).

Pain plays a very important role in this syndrome as well as in most post-viral affections, and indeed in Long-COVID19 (myalgias, paresthesia, headaches…). The fact that a small number of ME/CFS patients do not present any pain is definitely worthy of note, as these patients do not comply with the most widely used diagnostic criteria for Chronic Fatigue Syndrome Fukuda et al. (12). This highlights the need to follow homogeneous criteria and to identify clinical phenotypes that enable us to segment the analyses in a comparable way between different studies. Furthermore, the most frequent co-morbidity in both men and women was fibromyalgia syndrome. This means that in addition to a syndrome such as ME/CFS, which by itself is quite controversial, many patients (50 in our series) were also affected by Fibromyalgia, which by itself is also quite controversial. Both syndromes have superposed features, which makes the process of identifying and selecting patients for the study very difficult.

Anxiety and depression are relatively common with prevalence rates of 42.2 and 33.3%, respectively, and together form the second clustering criteria for the subgroup of patients without Fibromyalgia (29). Using the HAD questionnaire, we found that 36.9% of the patients were suffering from depression. This finding suggests an under diagnosis within our group, because only 14.28% had actually referred it. This shows how relevant is it to perform a differential diagnosis of “*depressive equivalents*” or masked depressions (30, 31) and supports the establishment of subgroups following the proposal of the American consensus group (13).

The second clustering criteria for the subgroup of patients with fibromyalgia is the presence of dysautonomic symptoms among others. These require specific assessment in specialized Dysautonomia Units. Impairment of the autonomic nervous system (ANS) has been proposed as a potential biomarker of ME/CFS (32, 33). A wide range of tests can be performed in these units. In our case, we propose the tilt table test, studies of small nerve fibers using cold stress tests recorded with a thermographic camera, the sudomotor function test, the micro-neurography test, evoked heat potentials, confocal microscopy of the cornea and skin biopsy. Some of these essential techniques seek to objectify the underlying autonomic dysfunction. In our group, there were only 16 cases (ten women and six men) that complied with the criteria for orthostatic intolerance syndromes (OI) in contrast with the high incidence of any dysautonomic features (circa 90%) referred by Robinson et al. (34). The identification of some of the forms of this syndrome, such as neurogenic orthostatic hypotension (NOH), orthostatic intolerance or postural orthostatic tachycardia syndrome (POTS), suggests the existence of a clinical phenotype or subgroup of ME/CFS patients with OI, as proposed in 2015 by the National Academy of Medicine of United States (13, 35). These aspects are also to be taken into account in Long-COVID19 patients. Our Dysautonomia Unit is evaluating 4–6 post-COVID19 patients every week in the last months. Just, since they reached the criterion for chronic fatigue (more than 6 months). Among other findings (pending of publication) we have found a remarkable similarity in the auto-antibodies profile for alfa and beta adrenergic receptors, muscarinic and angiotensin all of them linked mainly to POTS.

Almost all the patients had cognitive functions within normal range except for a small subgroup who presented slight cognitive deterioration. When asked to perform a task requiring sustained attention for 10 min in the Toulouse-Piéron test, we noticed a drop in the attention capacity (52.38% of patients), according to the findings obtained with other questionnaires. All of them support that the cognitive disorder present in this syndrome involve mainly the attention functions (36, 37).

Most of patients report poor quality of sleep and disorders such as changes in sleep patterns, unrefreshing sleep, etc. This is a serious problem, which could consolidate or exacerbate the symptoms (38). Only a small number of patients report good- quality sleep. ME/CFS has no specific pharmacological treatment. The available treatments are only symptomatic and the treatments for pain (non-steroidal anti- inflammatories, antidepressants) and anxiety (anxiolytics) play also a leading role for the management of the vicious circle of pain, anxiety and sleep disorders (39).

Functional autonomy is of enormous importance and can be measured using the Karnofsky scale. Our results are similar to other authors, with average scores of 54 for women and 62 for men (40). Few patients can continue working, but this aspect deserves a more detailed study because the difference between genders (nine women and only one man) is very substantial, compared to the average scores. On this point, it is important to bear in mind that most patients (64.28%) are in the midst of their working lives (35–51 years old). The direct and indirect costs of ME/CFS in America have been estimated about 18–24 billion dollars (41). Most patients affected by Long-COVID19 will probably heal in 6–12 months, but some of them (10–20%) could continue to have difficulties for years. Although the percentage is quite low, the absolute number of patients involved is high enough to create a challenge for our health and social services. The last wave of the pandemic will be its after-effects.

Some of the identified phenotypes appeared in the literature and our results only provide additional mathematical support for clinical evidences. A rigorous research approach to ME/CFS requires the use of statistical techniques (data mining) for a more precise diagnosis and phenotypic characterization (42). We have proposed a set of subgroups whose characteristics can serve as a basis for determining clinical-biological correlations (endophenotypes) in future research.

The pathology remains controversial, presenting changes in name and diagnostic criteria: ME, CFS or the last SEID (Systemic Exertional Intolerance Disease) (13); in fact, after a strict re-evaluation in our group all patients met both criteria Fukuda-1994 and Carruthers-2011. A single name and diagnostic criteria remain to be achieved. For instance, CFS-criteria (Fukuda-1994) do not rule out psychosomatic pathology (neuropsychiatric approach), instead ME (Carruthers-2011) exclude primary psychiatric pathology (neurological vision), but in this case a basic pathophysiological aspect, such as the existence of neuroinflammation, is not verified either. Therefore, the definition remains in a non-specificity neuro-immune-endocrine dysfunction or post-exertional neuroimmune malaise of complex objectification in any case. Nor has a standardized research methodology that allows data to be replicated. The investigation carried out propose a methodology to perform an extensive clinical evaluation of patients in order to establish clusters where be easier to understand the meaning of the biological findings.

In the current context of SARS Cov2 pandemic, the clinical status of Long COVID19 emerges (43) reporting fatigue, post-exertional malaise (PEM), cognitive problems (brain fog), etc. Various authors are orienting their reviews toward ME/CFS (44) looking indications for the future challenges (45). Patients with Long COVID19 can also be evaluated with our proposed battery of probes.

Limitations of the study include the small size of the patients group. We also proposed this fluctuating heterogeneous symptomatology should be assessed in a longitudinal study, with data collected in a central database and analyzed using big-data mining techniques.

## Conclusion

ME/CFS is a heterogeneous syndrome with multisystemic repercussions, classically linked to epidemic post-viral fatigue, which doctors must take into account in the aftermath of COVID-19. This syndrome has multiple clinical expressions and is associated with various co-morbidities. It is more prevalent amongst women than men.

Mathematical data mining can support clinical observation to provide a better understanding of the syndrome. A cluster analysis in our study group highlighted five different phenotypes. In three of them, the fibromyalgia was combined with varying degrees of dysautonomia, neuroendocrine or immunology features, while the other two phenotypes were free of fibromyalgia and presented high or low levels of anxiety-depression. There are no specific phenotypes for women or men, although some symptoms are more frequent in one or other gender.

It would also be very useful to have a centralized repository of clinical data, as a key tool for improving the design and implementation of new studies about this pathology.

## Data Availability Statement

The raw data supporting the conclusions of this article will be made available by the authors, without undue reservation.

## Ethics Statement

The studies involving human participants were reviewed and approved by Ethics Committee for Research involving Human Beings (CEISH - University of the Basque Country: act 80/2016 and 114/2019). The patients/participants provided their written informed consent to participate in this study.

## Author Contributions

IM has carried out the evaluation of the clinical cases. LA has carried out the statistical treatment of the data. PG, JG, and J-VL have made the clinical adjustments and discussion. All authors contributed to the article and approved the submitted version.

## Funding

This study has been funded by Instituto de Salud Carlos III through the project “PI20/01076” (Co-funded by European Regional Development Fund/European Social Fund “A way to make Europe”/“Investing in your future”).

## Conflict of Interest

The authors declare that the research was conducted in the absence of any commercial or financial relationships that could be construed as a potential conflict of interest.

## Publisher's Note

All claims expressed in this article are solely those of the authors and do not necessarily represent those of their affiliated organizations, or those of the publisher, the editors and the reviewers. Any product that may be evaluated in this article, or claim that may be made by its manufacturer, is not guaranteed or endorsed by the publisher.
